# O‐GlcNAcylation Mediated by OGA Activates NEK7/NLRP3 Pathway to Promote Pyroptosis in Parkinson's Disease

**DOI:** 10.1111/jcmm.70874

**Published:** 2025-10-09

**Authors:** Zhi Wang, Yue Liu, Lili Ma, Hongwei Sun, Ying Tang

**Affiliations:** ^1^ Department of Neurology The First Affiliated Hospital of Harbin Medical University Harbin China; ^2^ Key Laboratory of Hepatosplenic Surgery, Ministry of Education The First Affiliated Hospital of Harbin Medical University Harbin China

**Keywords:** NEK7, NLRP3, OGA, O‐GlcNAcylation, parkinson's disease, pyroptosis

## Abstract

Parkinson's disease (PD) is a neurodegenerative disorder characterised by pyroptosis. O‐GlcNAcylation, regulated solely by O‐GlcNAc transferase (OGT) and O‐GlcNAcase (OGA), has been shown to mitigate PD. This study aimed to investigate whether pyroptosis and PD pathogenesis are modulated by O‐GlcNAcylation. In PD model cells, O‐GlcNAc protein levels were downregulated, while OGA expression was upregulated. Knockdown of OGA significantly protected BV2 cells from LPS‐induced injury by inhibiting pyroptosis. Inhibition of OGA notably increased the O‐GlcNAc levels of NEK7. Furthermore, O‐GlcNAcylated NEK7 protein levels were significantly reduced by mutations at T170 or T172, whereas phosphorylated NEK7 protein levels were downregulated only by mutations at T172. Co‐immunoprecipitation (co‐IP) confirmed the endogenous interaction between NEK7 and NLRP3, which was weakened by OGA knockdown. In animal experiments, OGA deficiency significantly reduced motor dysfunctions and dopaminergic neurodegeneration in MPTP‐treated mice. OGT deficiency abolished the protective effects of OGA knockdown against MPTP‐induced injury. Additionally, OGT inhibition in OGA knockdown mice promoted pyroptosis. Collectively, these findings indicate that high OGA levels decrease O‐GlcNAcylation in PD, thereby promoting pyroptosis via the activation of the NEK7/NLRP3 pathway.

## 1 Introduction

Parkinson's disease (PD) is a prevalent neurodegenerative disorder characterised by the progressive loss of dopaminergic neurons in the substantia nigra [1]. The primary clinical symptoms include increased muscle tone, movement disorders and potential cognitive decline [2]. Currently, PD can only be managed through surgical interventions and pharmacological treatments, such as levodopa, levodopa synergists, dopamine receptor agonists and dopamine‐releasing agents; however, these treatments do not provide a cure [3]. Therefore, a deeper understanding of the pathogenesis of PD could facilitate the discovery of more effective therapies for the disease.

Research has shown that pyroptosis, as a programmed cell death mode of inflammatory cells, is mediated by the Gasdermin family and mainly regulated by inflammatory caspases, playing an important role in PD [4]. In PD mouse and cell models, the activation of the GSDMD‐mediated pyroptosis pathway is associated with the injury type activation of microglia and the death process of dopaminergic neurons. In addition, gene knockout of GSDMD or the use of the pyroptosis inhibitor disulfiram can inhibit pyroptosis and alleviate the pathological features of PD, demonstrating the potential therapeutic value of pyroptosis in PD pathology [5]. Therefore, research on pyroptosis helps to deepen our understanding of the pathogenesis of PD.

Pyroptosis is associated with the NLRP3 inflammasome complex and caspase‐1 activation. NEK7, as a multifunctional kinase, affects centrosome replication, mitochondrial regulation, intracellular protein transport, DNA repair and mitotic spindle assembly [1]. NEK7 has been reported as necessary for NLRP3 inflammasome activation in inflammasome activation [6]. The structures of human NEK7 and inactive NLRP3 show that NEK7 can bind to the leucine‐rich repeat sequence and nucleotide binding domain of NLRP3, indicating that NEK7 plays an important role in NLRP3 inflammasome activation [6]. Therefore, there is a direct correlation between pyroptosis and the NLRP3 inflammasome and NEK7, and the activity of NEK7 may be crucial for the activation of the NLRP3 inflammasome and pyroptosis process.

O‐GlcNAcylation is a post‐translational modification that shares common or similar modification sites with phosphorylation, specifically serine/threonine residues. Unlike phosphorylation, O‐GlcNAcylation is exclusively mediated by a pair of enzymes: O‐GlcNAc transferase (OGT) catalyses the addition of O‐GlcNAc, while O‐GlcNAcase (OGA) removes it from target proteins [7, 8]. This modification influences intracellular protein–protein interactions, stability, localisation and activity [9]. Lee et al. [10] demonstrated that interfering with the O‐GlcNAcylation of dopamine neurons alters cellular functions. Khidekel et al. [11] identified 25 O‐GlcNAc‐modified proteins in the brain, which are involved in regulating nerve signaling and synaptic plasticity. Consequently, O‐GlcNAcylation may have a potential relationship with neurological diseases, including PD. Previous studies have shown that targeting the NLRP3 inflammasome can alleviate PD [12, 13]. NEK7, an evolutionarily conserved group of protein kinases, binds to the NLRP3 leucine‐rich repeat domain in a kinase‐independent manner and plays a crucial role in the activation of the NLRP3 inflammasome [14]. However, whether the O‐GlcNAcylation of NEK7 regulates the NLRP3 inflammasome to influence PD remains to be elucidated.

In this study, we investigated whether O‐GlcNAcylation plays a key role in both in vivo and in vitro models of PD, and whether manipulating O‐GlcNAcylation can inhibit the pathology of PD. First, by examining the modification levels of O‐GlcNAc and its regulatory factors, OGT and OGA, in vitro, we found that abnormally high expression of OGA is associated with PD. In vivo, reducing OGA levels significantly ameliorated adverse indicators of PD. Additionally, we further explored the relationship between the NEK7–NLRP3 pathway and O‐GlcNAcylation in PD cell models. Collectively, our data suggest that inhibiting OGA to promote O‐GlcNAcylation can mitigate degeneration, dysfunction and motor deficits in models of PD.

## 2 Materials and Methods

### 2.1 Antibodies and Plasmids

The catalogue numbers and dilution ratios of all antibodies used in Immunocytochemistry (ICC) are listed as follows: anti‐IBA1 (#17198, 1:50, Cell Signalling Technology), anti‐GFAP (#80788, 1:200, Cell Signalling Technology), anti‐NEUN (ab177487, 1:100, Abcam), anti‐O‐GlcNAc (AG0461, 1:50, Beyotime), anti‐OGT (ab177941, 1:50, Abcam), anti‐OGA (23GB2270, 1:500, Invitrogen), goat anti‐rabbit IgG H&L (HRP) (ab6721, 1:1000, Abcam). The catalogue numbers and dilution ratios of all antibodies used in western blotting are listed as follows: anti‐IBA1 (#17198, 1:800, Cell Signalling Technology), anti‐GFAP (#80788, 1:800, Cell Signalling Technology), anti‐NEUN (#24307, 1:200, Cell Signalling Technology), anti‐O‐GlcNAc (#9875, 1:50, Cell Signalling Technology), anti‐OGT (#24083, 1:50, Cell Signalling Technology), anti‐OGA (#60406, 1:50, Cell Signalling Technology), anti‐Caspase‐1 (#24232, 1:1000, Cell Signalling Technology), anti‐cleaved‐Caspase‐1 (#89332, 1:1000, Cell Signalling Technology), anti‐GSDMD‐N (#39754, 1:1000, Cell Signalling Technology), anti‐cleaved‐GSDMD‐N (#34667, 1:1000, Cell Signalling Technology), anti‐NEK7 (#10054, 1:1000, Cell Signalling Technology), anti‐RL2 (ab93858, 1 μg/mL, Abcam), phospho‐NEK7 [Ser204, custom‐generated by Shanghai Guoyuan Biotechnology using a synthesised phosphopeptide corresponding to amino acids 201–212 of mouse NEK7 (YYMS204PERIHENG) as the antigen], anti‐GAPDH (#5174, 1:1000, Cell Signalling Technology), goat anti‐rabbit IgG H&L (HRP) (ab6721, 1:2000, Abcam). Expression vectors encoding shRNAs targeting OGA, driven by the U6 promoter and containing a puromycin‐resistant gene, were purchased from Sigma‐Aldrich. Lentiviral vectors expressing shRNAs against mouse OGA (Lv‐sh‐OGA) and a negative control (Lv‐sh‐NC) were obtained from GenePharma. The full‐length cDNA of mouse NEK7 and its C‐terminal and N‐terminal truncated mutants were amplified by PCR and subcloned into the pBudCE4.1‐3HA or pSEB‐3Flag vectors. Point mutations NEK7‐S52A, NEK7‐S100A, NEK7‐T170A and NEK7‐T172A were generated using the Hieff Mut Site‐Directed Mutagenesis Kit (YEASEN).

### 2.2 Cell Culture

BV2 microglial cells (American Type Culture Collection), murine ALTS1C1 astrocytes (BCRC60582, Hsin‐Chu, Taiwan) [15] and hippocampal neuronal HT‐22 cells (Chinese Academy of Sciences) were cultured in Dulbecco's Modified Eagle's Medium (DMEM/High) supplemented with 10% foetal bovine serum (Hyclone) and penicillin/streptomycin (Beyotime Biotechnology) in a 5% CO_2_ atmosphere at 37°C.

BV2 cells, ALTS1C1 cells and HT‐22 cells (2 × 10^5^ cells per well) were seeded in 6‐well plates and incubated with lipopolysaccharides (LPS, 100 ng/mL, L‐3129, Sigma‐Aldrich) for 24 h [16, 17].

To inhibit OGT expression, BV2 cells were pre‐treated with the OGT pharmacological inhibitor OSMI‐1 (5 μM, Sigma‐Aldrich) for 24 h prior to LPS stimulation in some experiments [18].

### 2.3 Generation of OGA Knockdown Cells

BV2 cells were stably transfected using Lipofectamine reagent (Invitrogen). Puromycin‐resistant cells selected by culturing in 1 μg/mL of puromycin‐containing medium were validated for the efficacy of knockdown via PCR in BV2 cells.

### 2.4 ICC

For ICC on BV2 cells of each group, 4% paraformaldehyde was used to fix the cells. Subsequently, fixed cells were washed with PBS and incubated in primary antibodies following the manufacturer's protocol. Following overnight primary antibody incubation, cells were washed with PBS, incubated in secondary antibodies for 75 min, and mounted onto slides using Fluoromount Aqueous Mounting Medium (MilliporeSigma). DAPI counterstaining was used to visualise the nuclei. Samples were visualised using an inverted fluorescence microscope (TE‐2000 U, Nikon). Image‐Pro Plus software 6.0 was used to count the co‐labelled cells.

### 2.5 Cell Viability

The viability of BV2 cells in each group was assessed using the CCK‐8 assay (Dojindo, Japan). After transfection and various treatments, BV2 cells were seeded into 96‐well plates and incubated with 10 μL of CCK‐8 solution. Following an additional 2‐h incubation at 37°C in a 5% CO_2_ atmosphere, the absorbance was measured at 450 nm using a microplate reader (BMG Labtech, Germany).

### 2.6 Detection of Lactate Dehydrogenase (LDH) Release

The LDH assay kit (Jiancheng, China) was used to determine the release of LDH. Briefly, BV2 cells (3 × 10^3^ cells per well) were inoculated into 96‐well plates, and LDH secretion was measured according to the manufacturer's instructions.

### 2.7 Enzyme‐Linked Immunosorbent Assay (ELISA)

Cell lysates of BV2 cells were used to evaluate the levels of IL‐1β (PI301, Beyotime Biotechnology) and IL‐18 (PI553, Beyotime Biotechnology) using commercially available ELISA detection kits according to the manufacturer's protocol.

### 2.8 DAPI/PI Double Staining

DAPI/PI double staining was performed to evaluate cell death in BV2 cells. BV2 cells were suspended in 1 mL of cell staining buffer and then stained with 5 μL of DAPI solution (C1005, Beyotime Biotechnology) and 5 μL of PI staining solution (ST511, Beyotime Biotechnology) at 4°C for 30 min. Finally, the stained cells were observed under a fluorescence microscope (TE‐2000 U, Nikon).

### 2.9 Western Blotting

Protein lysates from cells or tissues were extracted using cell lysis buffer (P0013, Beyotime Biotechnology) containing 1 mM phenylmethanesulfonyl fluoride (Beyotime Biotechnology). Equal volumes of protein samples were separated by 10% SDS‐PAGE and electrotransferred to PVDF membranes (IPVH00010, Merck Millipore). The membranes were probed with the indicated antibodies. Protein bands were visualised using SuperSignal West Pico Chemiluminescent Substrate Kits (Bio‐Rad). Quantification of the bands in Western blots was performed using Image‐Pro Plus software 6.0 (Media Cybernetics).

### 2.10 Co‐Immunoprecipitation (Co‐IP) and Detection of O‐GlcNAcylated NEK7

The endogenous binding of NEK7 with NLRP3 was detected by Co‐IP. BV2 cells were lysed in RIPA buffer (50 mM Tris–HCl, pH 7.4, 150 mM NaCl, 1% NP‐40, 0.5% sodium deoxycholate, 0.1% SDS) freshly supplemented with 1× cOmplete protease inhibitor cocktail (Roche) and 1 μM PUGNAc to preserve O‐GlcNAc modifications. Lysates (500 μg total protein) were pre‐cleared with 20 μL Protein A/G magnetic beads (Cat# 88802, Thermo Fisher) at 4°C for 30 min. Pre‐cleared supernatants were then incubated with 2 μg anti‐NEK7 rabbit polyclonal antibody or control normal rabbit IgG at 4°C for 3 h with gentle rotation. Subsequently, 20 μL fresh Protein A/G magnetic beads were added and incubation continued for 1 h at 4°C. Beads were washed three times with 1 mL ice‐cold RIPA buffer followed by one wash with 1 mL ice‐cold PBS. Bound proteins were eluted with 2× Laemmli sample buffer containing 100 mM DTT at 95°C for 5 min. Eluates were separated on 12% SDS‐PAGE gels and transferred to 0.22 μm PVDF membranes. Membranes were blocked with 5% BSA in TBST for 1 h at room temperature and then probed with anti‐O‐GlcNAc mouse monoclonal antibody RL2 overnight at 4°C. After washing, membranes were incubated with HRP‐conjugated anti‐mouse IgG for 1 h at room temperature. Immunoreactive bands were visualised using ECL substrate (Bio‐Rad). Membranes were stripped and re‐probed with anti‐GAPDH antibody as loading and IP normalisation controls. All experiments were performed in triplicate.

### 2.11 Animals and Experimental Design

All animal studies were approved by the Institutional Animal Care and Use Committee of The First Affiliated Hospital of Harbin Medical University and conducted in accordance with their guidelines. OGA expression was inhibited in 9‐week‐old male C57BL/6 mice via intracerebral stereotactic injection of lentivirus. Specifically, the mice were fasted for 4 h before the procedure and immobilised on a stereotactic brain locator (Stoelting) after inhalation of ether anaesthesia. Lentivirus (1 μL) was injected into the right substantia nigra at a rate of 0.25 μL/min using the following coordinates: 5.3 mm posterior to the bregma, 1.9 mm lateral to the midline and 7.5 mm below the skull. Post‐surgery, the mice were housed in a controlled environment with a 12‐h light/dark cycle and allowed to recover for 1 week. After recovery, the acute 1‐methyl‐4‐phenyl‐1,2,3,6‐tetrahydropyridine (MPTP) model was prepared to induce PD in 9‐week‐old male C57BL/6 mice. Mice were administered intraperitoneal injections of 30 mg/kg MPTP for five consecutive days at 24‐h intervals, following previously established protocols [19, 20]. All animals were randomly divided into eight groups: the C57BL/6 mice with PBS normal control (*n* = 6); the C57BL/6 mice with MPTP treatment (*n* = 6); the C57BL/6 mice with MPTP and Thiamet‐G (TMG) injection (50 mg/kg for 5 days) (*n* = 6) [21, 22]; the Lv‐sh‐NC mice with PBS normal control (*n* = 6); the Lv‐sh‐NC mice with MPTP treatment (*n* = 6); the Lv‐sh‐OGA mice with PBS normal control (*n* = 6); the Lv‐sh‐OGA mice with MPTP treatment (*n* = 6); the Lv‐sh‐OGA mice with MPTP and OSMI‐1 injection (10 mg/kg/day for 5 days) (*n* = 6) [18].

### 2.12 Behavioural Tests

A string test was performed to measure forelimb strength and coordination of the mice. The latent time, indicating the duration the mice held onto the bar, was recorded 24 h post‐final treatment [23]. Briefly, a narrow horizontal bar was fixed between two vertical supports, 20 cm above the floor. The mice were hung on the central point of the bar by their forepaws for 3 min, and the holding time was then measured.

For the rotarod test, the mice were trained 3 days before the test. On Day 4, the mice were placed on an accelerating rotarod cylinder, and the latency time of the animals was measured. The speed was slowly increased from 4 to 40 rpm within 5 min. A trial ended if the animal fell off the rungs or gripped the device and spun around for two consecutive revolutions without attempting to walk on the rungs.

The catalepsy test was carried out as previously described [24]. A horizontal bar with a diameter of 3 mm was placed 5 cm above the floor. The mice were positioned on the bar with their forepaws gripping the bar and their hindpaws resting on the floor. The time taken for the mice to move their forepaws off the bar was measured.

To measure hindlimb tension, the tails of the mice were suspended, and the position of the hindlimbs was observed for 10 s. Scoring was based on the position of the hindlimbs as follows: 0, both hindlimbs are completely detached from the abdomen; 1, one hindlimb pressed against the abdomen; 2, both hindlimbs partially closed toward the abdomen; 3, both hindlimbs were completely closed toward the abdomen.

### 2.13 Brain Tissue Sampling

After behavioural examination, the brains of the mice were harvested following intraperitoneal injection of 45 mg/kg pentobarbital sodium. The brain was divided into two identical hemispheres. One hemisphere was used to isolate the substantia nigra, with a portion fixed in 4% formaldehyde for histopathological studies. The other hemisphere was used to isolate both the substantia nigra and the striatum, which were then stored at −80°C for further analysis.

### 2.14 Determination of Striatal Dopamine Levels

The isolated striatum was homogenised in RIPA buffer. Homogenates were normalised using the Bradford assay, and the dopamine, 3,4‐dihydroxyphenylacetic acid (DOPAC), and homovanillic acid (HVA) levels in each sample were quantified using a high‐performance liquid chromatography system (Thermo Ultimate 3000). The separation was achieved using a C18 ion‐pair reverse‐phase analytical column (5 μm, 4.6 × 150 mm, Acclaim 120) at a flow rate of 0.6 mL/min. The mobile phase consisted of 8.65 mM heptane sulfonate, 0.27 mM EDTA, 13% acetonitrile, 0.43% triethylamine and 0.32% phosphoric acid.

### 2.15 Bioinformatic Analysis

The amino acid sequence of mouse NEK7 was analysed using the YinOYang server (http://www.cbs.dtu.dk/services/YinOYang/), which provides neural network predictions for O‐GlcNAc attachment sites in eukaryotic protein sequences [25].

### 2.16 Immunohistochemistry (IHC)

The brains were fixed in 4% paraformaldehyde and dehydrated with 30% sucrose solution until they sank to the bottom at 4°C. Brain sections were then stained with primary antibodies targeting tyrosine hydroxylase (Merck Millipore) and the appropriate secondary antibody. Nuclei were visualised using DAPI counterstaining. Images were acquired and processed using a microscope (TE‐2000 U, Nikon) and ZEN2011 software.

### 2.17 Statistical Analysis

SPSS version 26.0 (IBM, USA) was used to analyse all data. Continuous variables are presented as mean ± standard deviation (SD). Inter‐group comparisons were made using two‐tailed independent samples *t*‐tests, while intra‐group comparisons were made using two‐tailed paired sample *t*‐tests. For all tests, a *p*‐value < 0.05 was considered statistically significant.

## 3 Results

### 3.1 Downregulation of O‐GlcNAcylation Mediated by OGA Induced PD In Vitro and In Vivo

**FIGURE 1:**
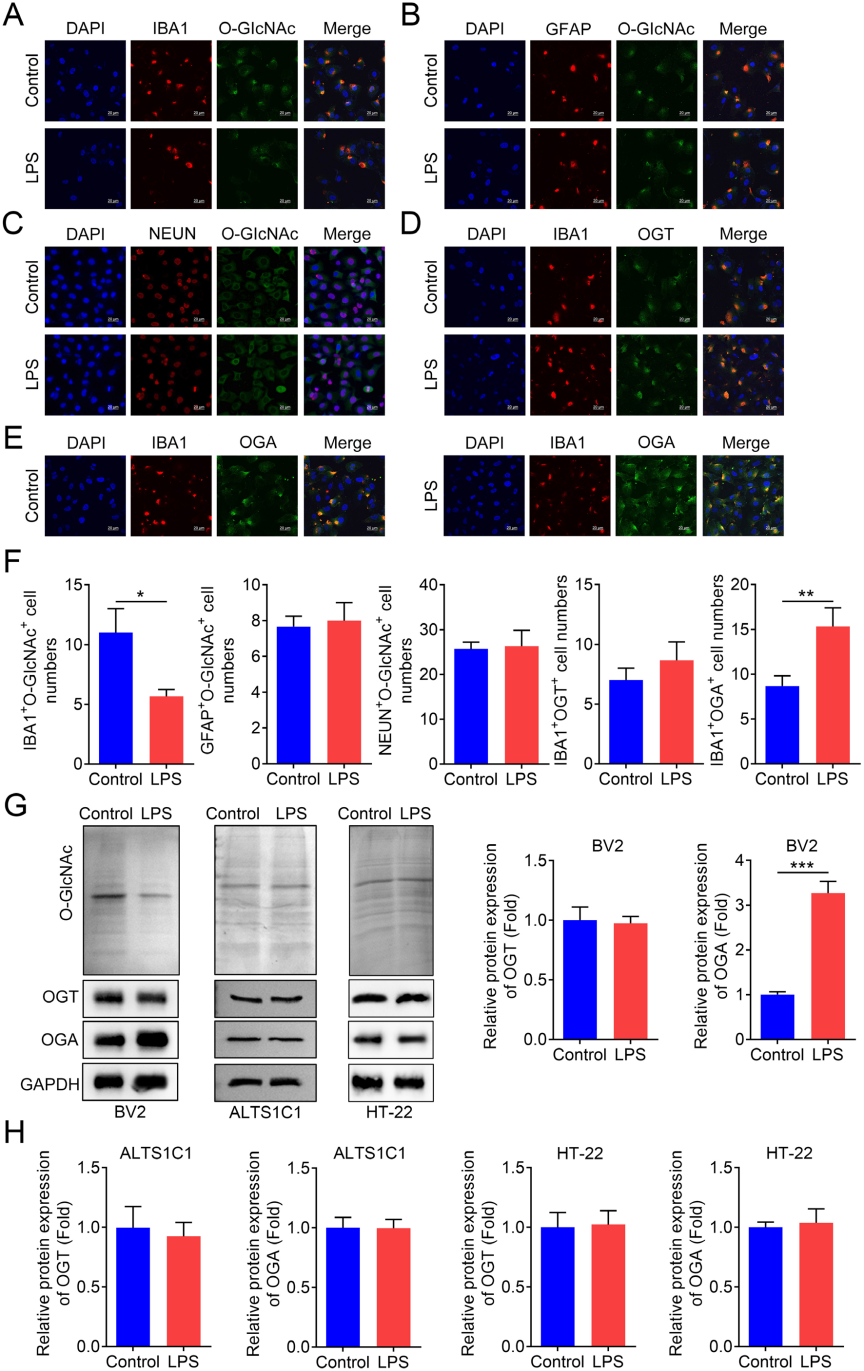
Expression of levels of typical markers, O‐GlcNAc, OGT and OGA in three PD model cells. (A—E) Representative images of typical markers, O‐GlcNAc, OGT and OGA of microglia, astrocyte and neuronal cells identified by ICC method. (F) Quantitative analysis of IBA1, GFAP, NEUN, O‐GlcNAc, OGT and OGA levels in microglia, astrocyte and neuronal cells identified by ICC method, *n* = 3. Quantification performed on a per‐cell basis using ImageJ software, normalised to DAPI‐positive cell numbers. (G—H) Representative images and quantitative analysis of OGT and OGA of microglia, astrocyte, and neuronal cells identified by western blotting method, *n* = 3. *p < 0.05, **p < 0.01.

**FIGURE 2:**
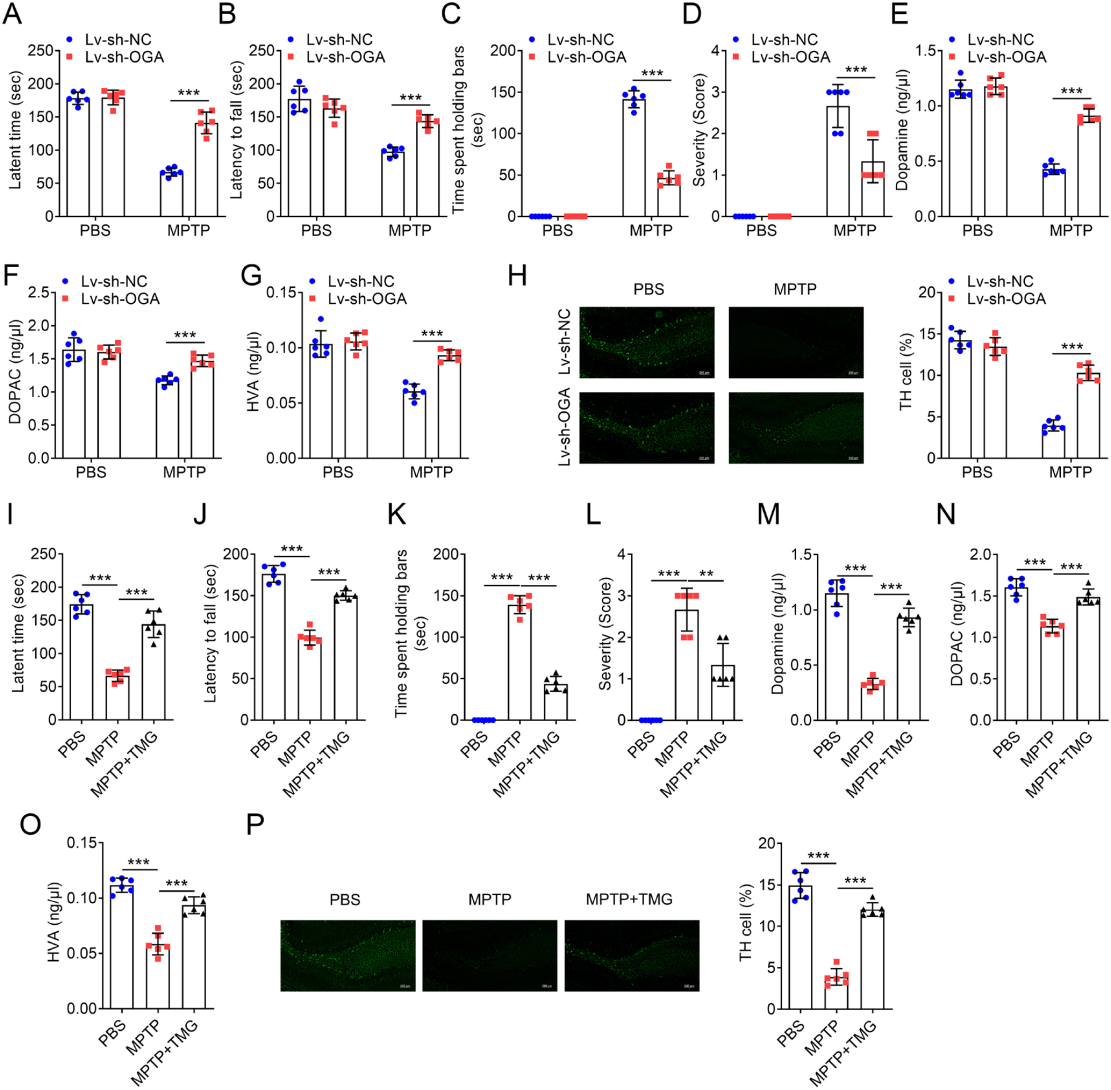
OGA deficiency attenuates motor dysfunctions and dopaminergic neuronal loss in MPTP‐treated mice. (A) The latent time to hold the bar in the string test 24 h post the final treatment of Lv‐sh‐NC and Lv‐sh‐OGA mice, *n* = 6. (B) The latency to fall by the rotarod test 1 h post the final treatment of Lv‐sh‐NC and Lv‐sh‐OGA mice, *n* = 6. (C) The latent time to hold the bar in the catalepsy test 1 h post the final treatment of Lv‐sh‐NC and Lv‐sh‐OGA mice, *n* = 6. (D) The severity of hindlimb clasping in mice 1 h post the final treatment of Lv‐sh‐NC and Lv‐sh‐OGA mice, *n* = 6. (E–G) Quantification of striatal dopamine, DOPAC, and HVA levels of Lv‐sh‐NC and Lv‐sh‐OGA mice as determined by high performance liquid chromatography system, *n* = 6. (H) Representative IHC image and quantification of relative TH‐positive cells of the fixed brain sections of Lv‐sh‐NC and Lv‐sh‐OGA mice after staining with anti‐TH antibody (green). Scale bars, 200 μm, *n* = 6. (I) The latent time to hold the bar in the string test 24 h post the final treatment of C57BL/6 mice treated with TMG and/or MPTP, *n* = 6. (J) The latency to fall by the rotarod test 1 h post the final treatment of C57BL/6 mice treated with TMG and/or MPTP, *n* = 6. (K) The latent time to hold the bar in the catalepsy test 1 h post the final treatment of C57BL/6 mice treated with TMG and/or MPTP, *n* = 6. (L) The severity of hindlimb clasping in mice 1 h post the final treatment of C57BL/6 mice treated with TMG and/or MPTP, *n* = 6. (M–O) Quantification of striatal dopamine, DOPAC and HVA levels of C57BL/6 mice treated with TMG and/or MPTP as determined by high performance liquid chromatography system, *n* = 6. (P) Representative IHC image and quantification of relative TH‐positive cells of the fixed brain sections of C57BL/6 mice treated with TMG and/or MPTP after staining with anti‐TH antibody (green). Scale bars, 200 μm, *n* = 6. ***p < 0.001.

To investigate the functional importance of O‐GlcNAcylation in vitro, we first assessed the O‐GlcNAc levels in three PD model cell lines. ICC was used to determine the expression of typical markers for microglia, astrocytes and neuronal cells. The results showed that the levels of IBA1, GFAP and NEUN were significantly elevated in the respective cell types (Figure 1A–C,F). Concurrently, the fluorescence intensity of IBA^+^O‐GlcNAc^+^ in BV2 microglia significantly decreased after LPS treatment, while LPS treatment had no significant effect on the fluorescence intensity of O‐GlcNAc^+^ in the other two types of cells (Figure 1A–C,F). OGA is responsible for removing O‐GlcNAc modifications, while OGT is responsible for adding O‐GlcNAc modifications, both of which jointly regulate the O‐GlcNAcylation status of proteins. We further found that LPS significantly increased the expression level of OGA but had no statistical effect on OGT levels (Figure 1D–F). We detected consistent results with the ICC method using western blot, indicating that LPS decreased O‐GlcNAc levels in microglia by affecting the protein expression of OGA (Figure 1G,H). In addition, compared with the PBS control group, the MPTP‐induced PD model group also had higher levels of O‐GlcNAc in the mouse brain tissues (Figure S1). To further explore the in vivo significance, Lv‐sh‐NC and Lv‐sh‐OGA mice were treated with MPTP to induce PD. MPTP treatment significantly reduced the latent time in the string test (Figure 2A), latency to fall by the rotarod test (Figure 2B), indicating impaired motor activity. Similarly, MPTP‐treated mice exhibited a marked increase in cataleptic response (Figure 2C). The severity of hindlimb clasping was also significantly increased in MPTP‐treated mice compared to the PBS control groups (Figure 2D). In the study of PD, the levels of DA, DOPAC and HVA are widely used to evaluate the functional status and pathological changes of dopaminergic neurons. Additionally, a significant reduction in striatal dopamine levels was observed in the MPTP‐treated groups (Figure 2E–G). The effects of MPTP were more pronounced in the Lv‐sh‐NC groups. Following MPTP administration, the levels of TH in dopaminergic neurons were examined. As expected, MPTP treatment led to a significant reduction in TH‐positive dopaminergic neurons in Lv‐sh‐NC mice (Figure 2H). Notably, the MPTP‐induced loss of dopaminergic neurons was significantly mitigated in OGA‐deficient mice (Figure 2A–H). Our data suggest that MPTP administration induced motor dysfunctions and dopaminergic neuronal loss in C57BL/6 mice. Treatment with TMG, an OGA inhibitor, significantly reversed the effects of MPTP (Figure 2I–P). These findings indicate that OGA‐mediated O‐GlcNAcylation is closely associated with PD both in vitro and in vivo, and that inhibition of OGA contributes to the alleviation of PD progression.

## 4 Inhibition of OGA Alleviated Pyroptosis in PD Model Cells

**FIGURE 3:**
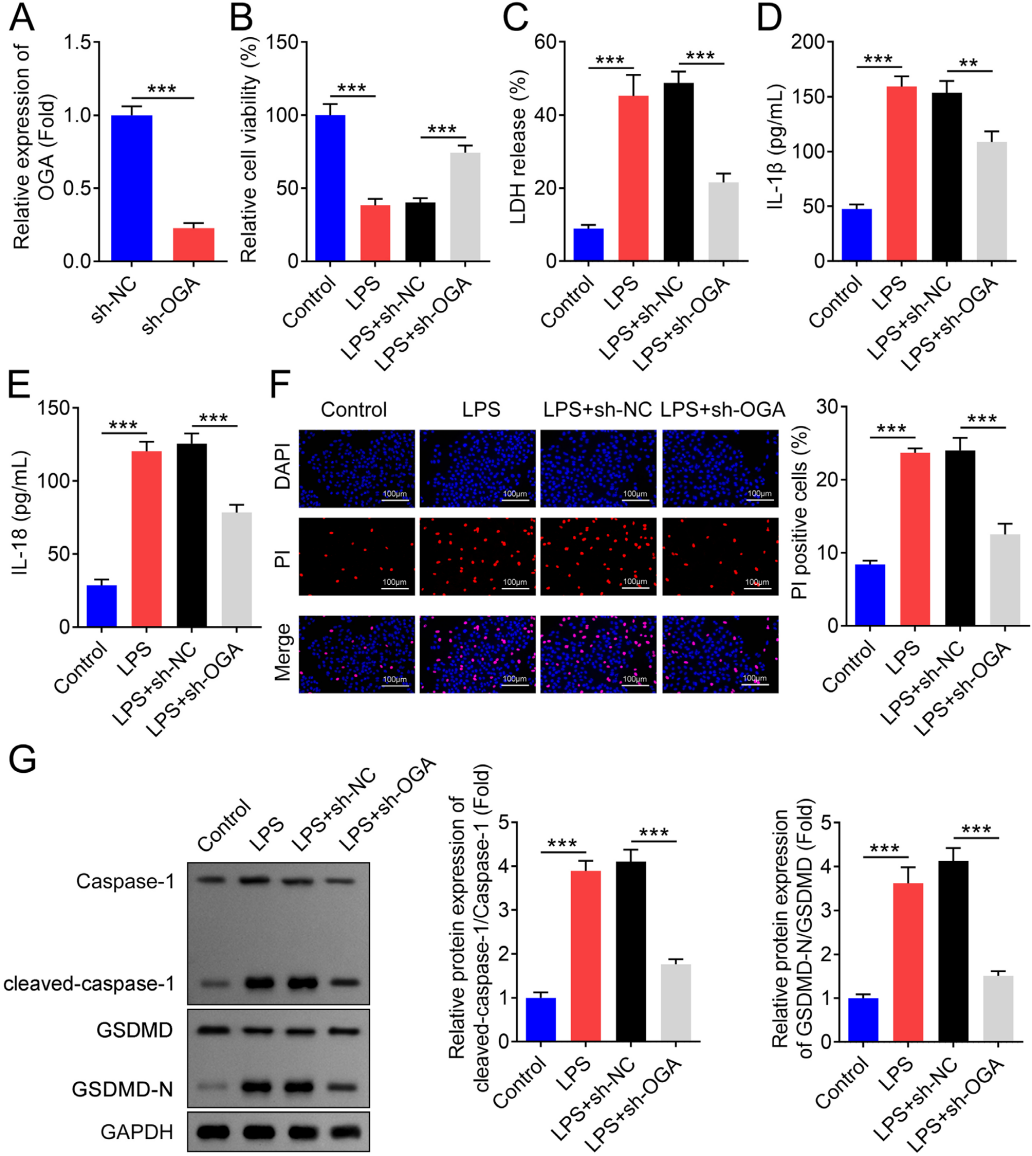
Knockdown of OGA inhibits pyroptosis of LPS‐treated BV2 cells. (A) The relative expression of BV2 cells transfected with sh‐OGA plasmids, *n* = 3. (B) Relative cell viability of BV2 cells transfected with LV‐sh‐OGA plasmids and/or LPS treatment was accessed by CCK‐8 assay., *n* = 3. (C—E) LDH, IL‐1β and IL‐18 concentration of BV2 cells transfected with LV‐sh‐OGA plasmids and/or LPS treatment were evaluated by commercial kits, *n* = 3. (F) Cell death was evaluated by DAPI/PI staining, and the number of BV2 cells transfected with LV‐sh‐OGA plasmids and/or LPS treatment was quantified, *n* = 3. (G) Representative images of the cleaved‐caspase‐1 and cleaved‐GSDMD‐N proteins and the quantification analysis in BV2 cells transfected with LV‐sh‐OGA plasmids and/or LPS treatment, *n* = 3. ***p < 0.001.

Pyroptosis has been reported to play a key role in PD [26]. Treatment with LPS significantly decreased the viability of BV2 microglial cells (Figure S2A) and increased the secretion of LDH, IL‐1β and IL‐18 (Figure S2B–D). Additionally, the number of dead BV2 cells increased upon LPS administration, as confirmed by DAPI/PI staining (Figure S2E). Western blot analysis revealed that the levels of cleaved caspase‐1 and cleaved GSDMD‐N were significantly upregulated in the LPS treatment group (Figure S2F). To investigate whether O‐GlcNAcylation regulates pyroptosis in PD, we inhibited OGA expression in BV2 cells. Successful inhibition of OGA was confirmed (Figure 3A). Following OGA inhibition, the viability of BV2 cells was significantly improved, while LPS‐induced increases in LDH, IL‐1β and IL‐18 secretion, as well as the number of dead cells, were attenuated (Figure 3B–F). Furthermore, inhibition of OGA significantly downregulated the protein levels of cleaved caspase‐1 and cleaved GSDMD‐N in LPS‐treated BV2 cells (Figure 3G). These findings suggest that OGA inhibition can mitigate LPS‐induced pyroptosis in BV2 cells, highlighting the potential therapeutic role of O‐GlcNAcylation in PD.

## 5 OGA Deficiency Increased the O‐GlcNAcylation of NEK7 Protein and Weakened the NEK7/NLRP3 Interaction

**FIGURE 4:**
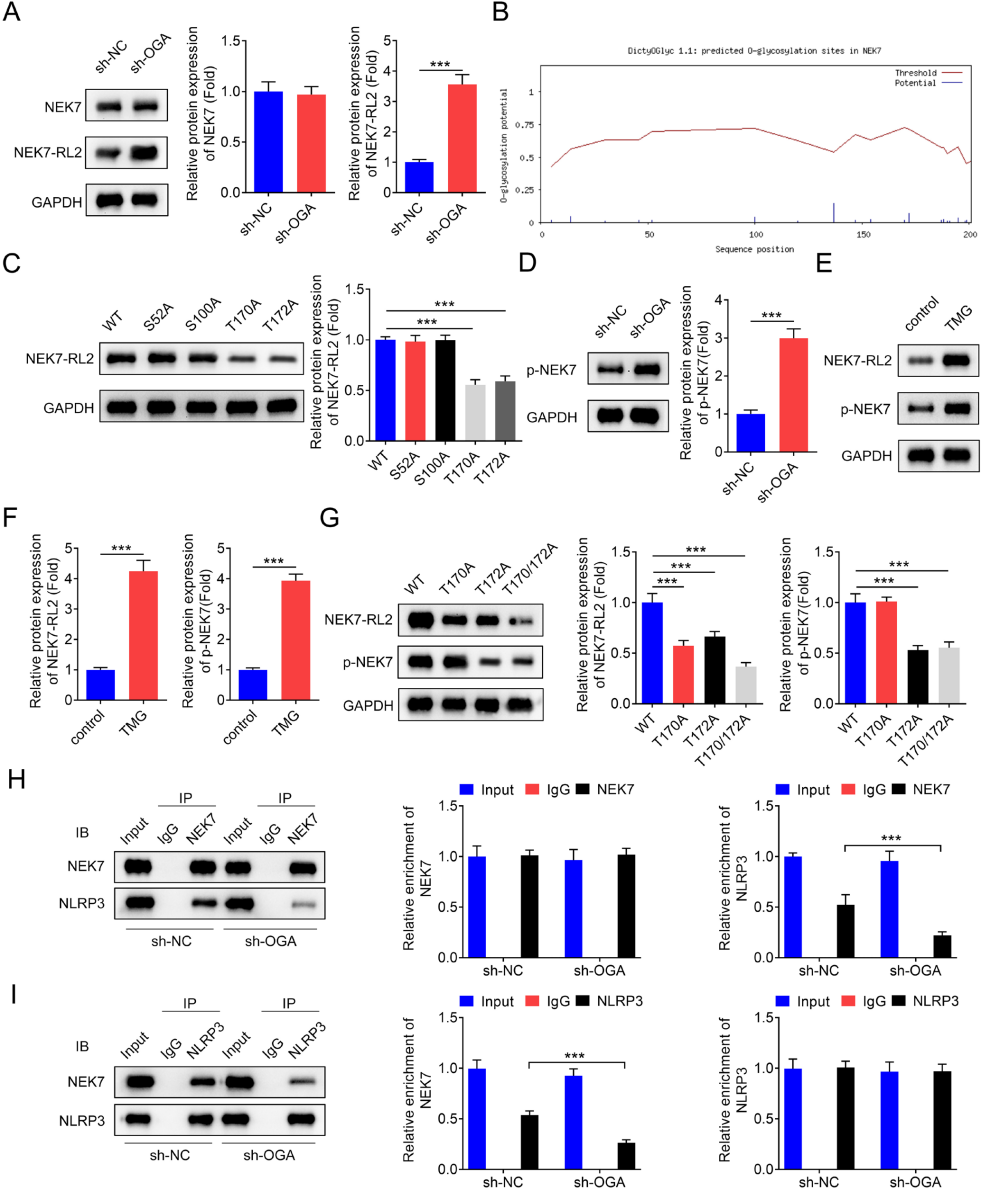
OGA deficiency increased the O‐GlcNAcylation of NEK7 protein and weakened the NEK7/NLRP3 interaction. (A) Representative images of the NEK7 and O‐GlcNAcylated NEK7 proteins in BV2 cells after knockdown of OGA and the quantification analysis, *n* = 3. (B) The potential O‐GlcNAc sites of NEK7 predicted by the YinOYang server (http://www.cbs.dtu.dk/services/YinOYang/). (C) Representative images of the O‐GlcNAcylated NEK7 proteins with different mutation in BV2 cells and the quantification analysis, *n* = 3. (D) Representative images of the phosphorylated NEK7 proteins in BV2 cells with OGA knockdown and the quantification analysis, *n* = 3. (E—F) Representative images of the phosphorylated NEK7 and O‐GlcNAcylated NEK7 proteins in BV2 cells treated with TMG and the quantification analysis, *n* = 3. (G) Representative images of the phosphorylated NEK7 and O‐GlcNAcylated NEK7 proteins in BV2 cells with different mutation and the quantification analysis, *n* = 3. (H—I) Co‐IP method was performed to verify the endogenous interaction relationship between NEK7 and NLRP3, *n* = 3. ***p < 0.001.

Given that the NEK7/NLRP3 signalling pathway modulates pyroptosis in various diseases [1, 27], investigating the O‐GlcNAcylation of the NEK7 protein in PD is warranted. NEK7 was immunoprecipitated from BV2 lysates and then immunoblotted with RL2 to detect O‐GlcNAc modification. The total protein levels of NEK7 were not significantly affected by OGA inhibition; however, the O‐GlcNAc levels of NEK7 were notably increased (Figure 4A). As shown in Figure 4B, the exact O‐GlcNAc site could not be determined due to the presence of multiple potential O‐GlcNAc sites in the NEK7 protein. To identify the specific sites, we mutated each of the first four sites with the highest potential for O‐GlcNAcylation. Western blot analysis revealed that mutations at T170 and T172 significantly downregulated the O‐GlcNAc level of NEK7 (Figure 4C). Furthermore, inhibition of OGA upregulated the protein levels of phosphorylated NEK7 (Figure 4D). Treatment with TMG, an OGA inhibitor, also increased the protein levels of both phosphorylated NEK7 and O‐GlcNAcylated NEK7 (Figure 4E,F). Additionally, mutations at T170 or T172 significantly downregulated the O‐GlcNAcylated NEK7 protein levels, while phosphorylated NEK7 protein levels were downregulated only by mutations at T172 (Figure 4G). To further explore the endogenous interaction between NEK7 and NLRP3, co‐IP was performed. The results confirmed the interaction between NEK7 and NLRP3. Notably, knockdown of OGA, which increased O‐GlcNAcylated NEK7 protein levels, significantly weakened the interaction between NEK7 and NLRP3 (Figure 4H,I). These findings suggest that O‐GlcNAcylation of NEK7 at specific sites, particularly T170 and T172, plays a crucial role in modulating the NEK7/NLRP3 signalling pathway and pyroptosis in PD.

## 6 Inhibition of OGT Reversed the Effects of OGA Deficiency on PD Model Cells and Animals

**FIGURE 5:**
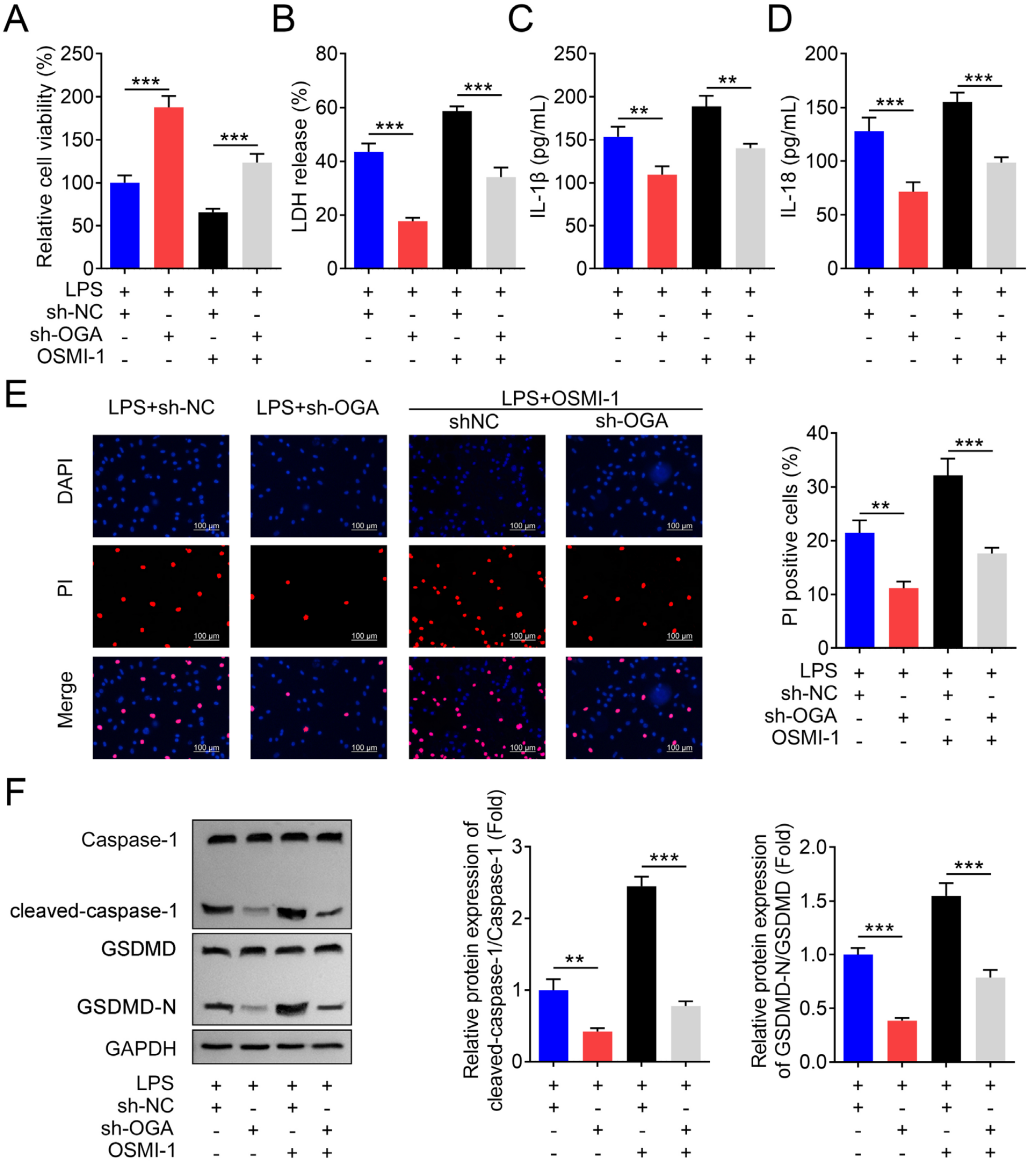
Inhibition of OGT reversed the effects of OGA deficiency on PD model cells. (A) Relative cell viability of BV2 cells with OSMI‐1 treatment before and after OGA inhibition was accessed by CCK‐8 assay, *n* = 3. (B—D) LDH, IL‐1β, and IL‐18 concentration of OSMI‐1‐treated BV2 cell lysis before and after OGA inhibition were evaluated by commercial kits, *n* = 3. (E) Cell death was evaluated by DAPI/PI staining, and the number of cells before and after OGA inhibition was quantified, *n* = 3. (F) Representative images of the cleaved‐caspase‐1 and cleaved‐GSDMD‐N proteins and the quantification analysis in BV2 cells before and after OGA inhibition, *n* = 3. **p < 0.01, ***p < 0.001.

**FIGURE 6:**
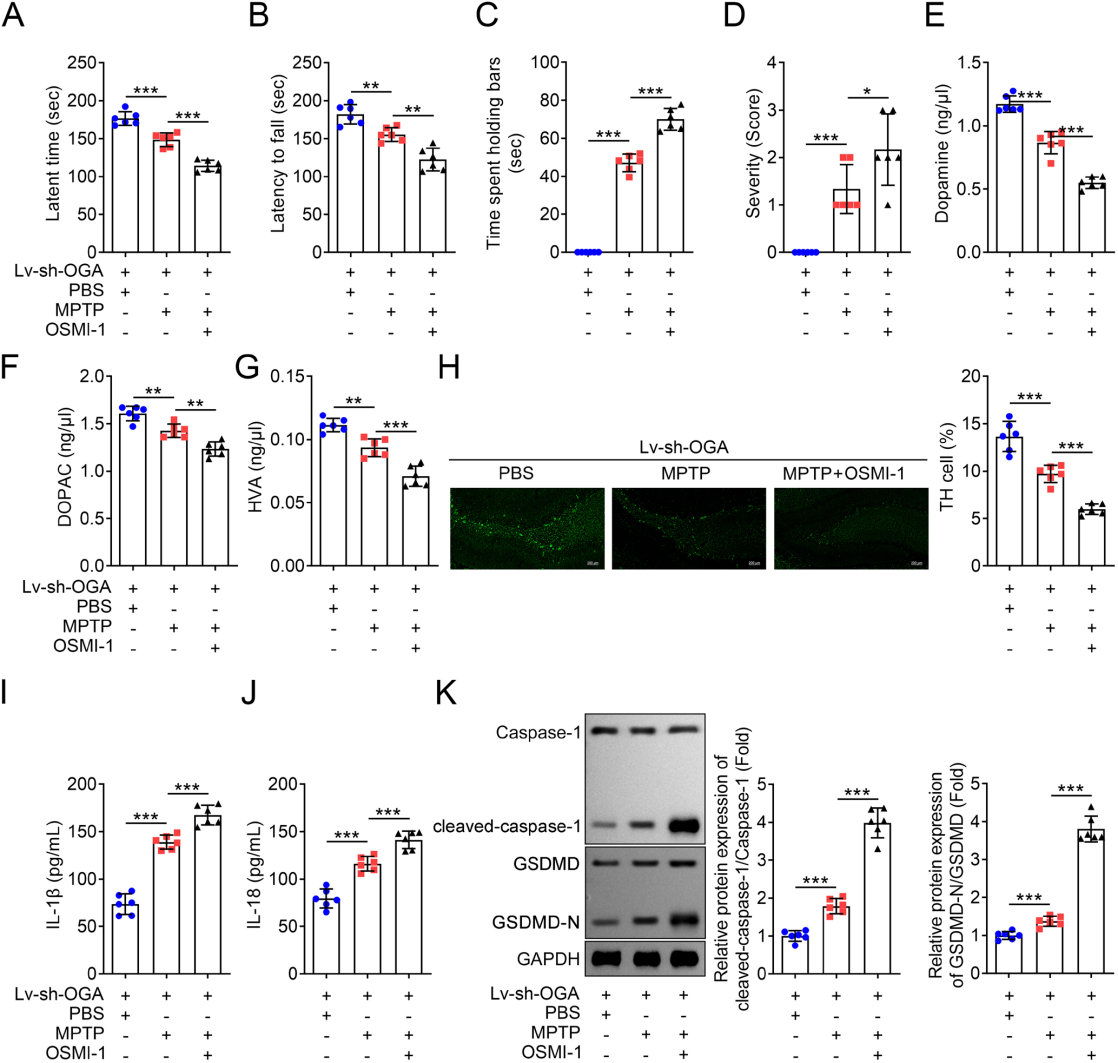
Inhibition of OGT reversed the effects of OGA deficiency on PD model animals. (A) The latent time to hold the bar in the string test 24 h post the final treatment of Lv‐sh‐OGA mice, *n* = 6. (B) The latency to fall by the rotarod test 1 h post the final treatment of Lv‐sh‐OGA mice, *n* = 6. (C) The latent time to hold the bar in the catalepsy test 1 h post the final treatment of Lv‐sh‐OGA mice, *n* = 6. (D) The severity of hindlimb clasping in mice 1 h post the final treatment of Lv‐sh‐OGA mice, *n* = 6. (E—G) Quantification of striatal dopamine, DOPAC and HVA levels of Lv‐sh‐OGA mice as determined by high performance liquid chromatography system, *n* = 6. (H) Representative IHC image and Quantification of relative TH‐positive cells of the fixed brain sections of Lv‐sh‐OGA mice after staining with anti‐TH antibody (green). Scale bars, 200 μm, *n* = 6. (I—J) IL‐1β and IL‐18 concentration were evaluated by commercial kits, *n* = 6. (K) Representative images of the cleaved‐caspase‐1 and cleaved‐GSDMD‐N proteins and the quantification analysis, *n* = 6. *p < 0.05, **p < 0.01, ***p < 0.001.

Since O‐GlcNAcylation is exclusively mediated by OGT and OGA, as previously reported [10, 28], inhibition of OGT may lead to an increase in OGA activity. As shown in Figure 5A, after knockdown of OGA, the viability of BV2 cells treated with the OGT pharmacological inhibitor OSMI‐1 was significantly decreased compared to untreated BV2 cells (Figure 5A). Additionally, the secretion of LDH, IL‐1β and IL‐18 was markedly elevated in BV2 cells with inhibited OGT (Figure 5B–D). Furthermore, cell death (Figure 5E) and pyroptosis were significantly promoted after OGT suppression (Figure 5F). In vivo experiments also demonstrated that suppression of OGT exacerbated MPTP‐induced injury. This was evident from the significant impairment of motor activity (Figure 6A,B), increased cataleptic response (Figure 6C), increased severity of hindlimb clasping (Figure 6D), and a reduction in striatal dopamine levels (Figure 6E–G). Moreover, MPTP‐induced pyroptosis was significantly enhanced by OSMI‐1 treatment (Figure 6H–K). These findings underscore the critical role of OGT in modulating pyroptosis and suggest that OGT inhibition exacerbates the pathological processes associated with PD.

## 7 Discussion

O‐GlcNAcylation is essential for the regulation of nerve function in various neurodegenerative diseases, and its levels fluctuate during both normal and pathological ageing. Lee et al. [29] reported that elevated O‐GlcNAcylation significantly mitigated PD pathology in dopaminergic neurons, including the abnormal aggregation of alpha‐synuclein and neuronal death. Increased O‐GlcNAc levels can alleviate the pathology and associated physiological symptoms of PD [29]. Previous studies have demonstrated that the activation of microglia, astrocytes and neuronal cells is a central process in neuroinflammation, which is a major contributor to the pathogenesis of neurodegenerative diseases [30]. In this study, LPS administration was used to induce PD in vitro in microglia, astrocytes and neuronal cells. Our data indicated that changes in O‐GlcNAcylation were most pronounced in BV2 microglial cells. Moreover, OGA was significantly upregulated in LPS‐treated BV2 microglial cells. As previously reported, dopamine neuron‐specific OGA knockout mice exhibited enhanced dopamine release in the striatal dopaminergic terminals [10]. Our findings align with these previous reports [23]. MPTP treatment significantly induced dopaminergic neuronal loss and subsequent motor deficits. The MPTP‐induced injury was markedly attenuated by the OGA inhibitor TMG and by genetic OGA knockdown. These results highlight the critical role of OGA‐mediated O‐GlcNAcylation in the pathogenesis of PD and suggest that targeting OGA may be a promising therapeutic strategy for mitigating PD progression.

Pyroptosis may play a significant role in the occurrence and development of neurodegenerative diseases, and the abnormal expression of inflammatory mediators can either promote or inhibit the pathological processes of these diseases [13, 31]. Caspase‐1‐dependent classical pyroptosis can catalyse the maturation and secretion of IL‐1β and IL‐18 into the extracellular space, further damaging dopaminergic neurons and contributing to the progression of PD [13]. In vitro experiments have confirmed that MPTP stimulation of primary microglia can promote the activation and maturation of caspase‐1 and IL‐1β. Studies have shown that knocking out NLRP3 or caspase‐1 can inhibit the progression of MPTP‐induced PD in mice [23, 32, 33]. Therefore, targeted regulation of pyroptosis can be an effective strategy for preventing and treating neurodegenerative diseases. NEK7 is a newly identified upstream regulator of NLRP3. NEK7 regulates NLRP3 signalling by binding to the LRR domain [14]. NEK7 is involved in the aggregation process of the inflammasome protein complex, and its deletion can specifically block the activation of the NLRP3 inflammasome and the release of IL‐1β, thereby modulating pyroptosis [1]. Moreover, O‐GlcNAcylation modifies the same or similar sites as phosphorylation, specifically serine/threonine residues. Due to this similarity, there is competition between these modifications. Many proteins modified by O‐GlcNAcylation are also known to be phosphorylated [7, 34]. We hypothesised that NEK7/NLRP3‐mediated pyroptosis may be regulated by O‐GlcNAcylation, thereby affecting its phosphorylation. In this study, LPS treatment induced pyroptosis, and inhibition of OGA suppressed this process. O‐GlcNAcylated NEK7 levels were increased by OGA inhibition, with the modification sites identified as T170 and T172. Additionally, phosphorylation at T172 was influenced by O‐GlcNAcylation modification. Interestingly, OGA inhibition weakened the interaction between NEK7 and NLRP3. Therefore, OGA inhibition enhances the O‐GlcNAcylation of NEK7, which in turn inhibits phosphorylation at the T172 site, thereby suppressing pyroptosis. These findings suggest that O‐GlcNAcylation of NEK7 is a critical regulatory mechanism in the NEK7/NLRP3 signalling pathway and may represent a novel therapeutic target for the treatment of PD.

Since OGT and OGA regulate O‐GlcNAcylation in opposing manners, we hypothesised that inhibition of OGT might function similarly to overexpression of OGA in regulating PD both in vitro and in vivo. Indeed, OGT inhibition induced by OSMI‐1 reversed the effects of OGA knockdown, thereby alleviating pyroptosis and inhibiting dopaminergic neuronal loss and the subsequent motor deficits. These findings suggest that the balance between OGT and OGA is crucial for maintaining proper O‐GlcNAcylation levels, which in turn modulates the inflammatory and neurodegenerative processes associated with PD. Thus, targeting OGT and OGA may offer potential therapeutic strategies for mitigating the progression of PD.

In this study, we recognised some limitations in the experimental design. Our study did not fully explore how OGA deficiency affects the interactions between microglia, astrocytes and neurons. Future research will use co‐culture systems or conditional medium transfer experiments to gain a deeper understanding of the communication dynamics between these cells, which will help reveal the role of O‐GlcNAcylation in regulating neuroinflammation and neurodegeneration. Furthermore, although LPS treatment is a widely used model for inducing neuroinflammation, it may not fully represent the pathophysiology of PD. PD mainly involves the degeneration of dopaminergic neurons, while LPS treatment simulates more inflammatory responses. Therefore, we plan to use inducers such as 6‐hydroxydopamine, rotenone or MPP+ in future research that are closer to the pathological mechanisms of oxidative stress and mitochondrial dysfunction in PD [35, 36, 37].

## 8 Conclusion

In summary, OGA is aberrantly elevated in LPS‐treated BV2 cells, and inhibition of OGA significantly protects BV2 cells from pyroptosis injury. OGA deficiency markedly alleviates motor deficits and dopaminergic neuronal loss in PD animal models. Furthermore, enhancing O‐GlcNAcylation through OGA knockdown suppresses the phosphorylation of NEK7, thereby weakening the interaction between NEK7 and NLRP3. These findings highlight the therapeutic potential of targeting OGA in the treatment of PD.

## Author Contributions

**Zhi Wang:** supervision (equal), validation (equal), visualization (equal), writing – original draft (equal), writing – review and editing (equal). **Yue Liu:** data curation (equal), formal analysis (equal), investigation (equal), resources (equal), writing – review and editing (equal). **Lili Ma:** data curation (equal), formal analysis (equal), investigation (equal), resources (equal), writing – review and editing (equal). **Hongwei Sun:** conceptualization (equal), methodology (equal), writing – review and editing (equal). **Ying Tang:** conceptualization (equal), methodology (equal), writing – review and editing (equal).

## Ethics Statement

All animal studies were approved by The First Affiliated Hospital of Harbin Medical University and performed in accordance with the Institutional Animal Care and Use Committee.

## Consent

The authors have nothing to report.

## Conflicts of Interest

The authors declare no conflicts of interest.

## Supporting information


**Figure S1:** The levels of O‐GlcNAc in mouse brain tissues in both PBS and MPTP group evaluated by western blotting method.
**Figure S2:** LPS treatment induces pyroptosis of BV2 cells.

## Data Availability

The datasets used and/or analysed during this study are available from the corresponding author on reasonable request.
