# Supplementary material for: O‐GlcNAcylation Mediated by OGA Activates NEK7/NLRP3 Pathway to Promote Pyroptosis in Parkinson's Disease
Source: J Cell Mol Med. 2025 Oct 9;29(19):e70874. doi: 10.1111/jcmm.70874 (PMC12510429; doi:10.1111/jcmm.70874)

Supplementary Figure 1. The levels of O-GlcNAc in mouse brain tissues in both PBS and MPTP group evaluated by western blotting method.


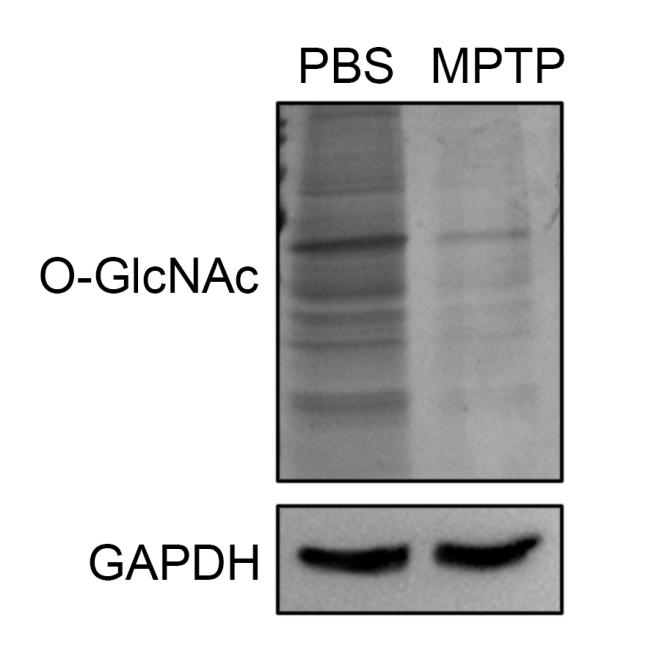


Supplementary Figure 2. LPS treatment induces pyroptosis of BV2 cells.

A Relative cell viability of BV2 cells with LPS treatment was accessed by CCK-8 assay., n=3

B - D LDH, IL-1β, and IL-18 concentration of LPS-treated BV2 cell lysis were evaluated by commercial kits, n=3.

E Cell death was evaluated by DAPI/PI staining and the number of cells was quantified, n=3.

F Representative images of the cleaved-caspase-1, cleaved-GSDMD-N, O-GlcNAc, OGT, and OGA proteins and the quantification analysis, n=3.

****p*<0.001


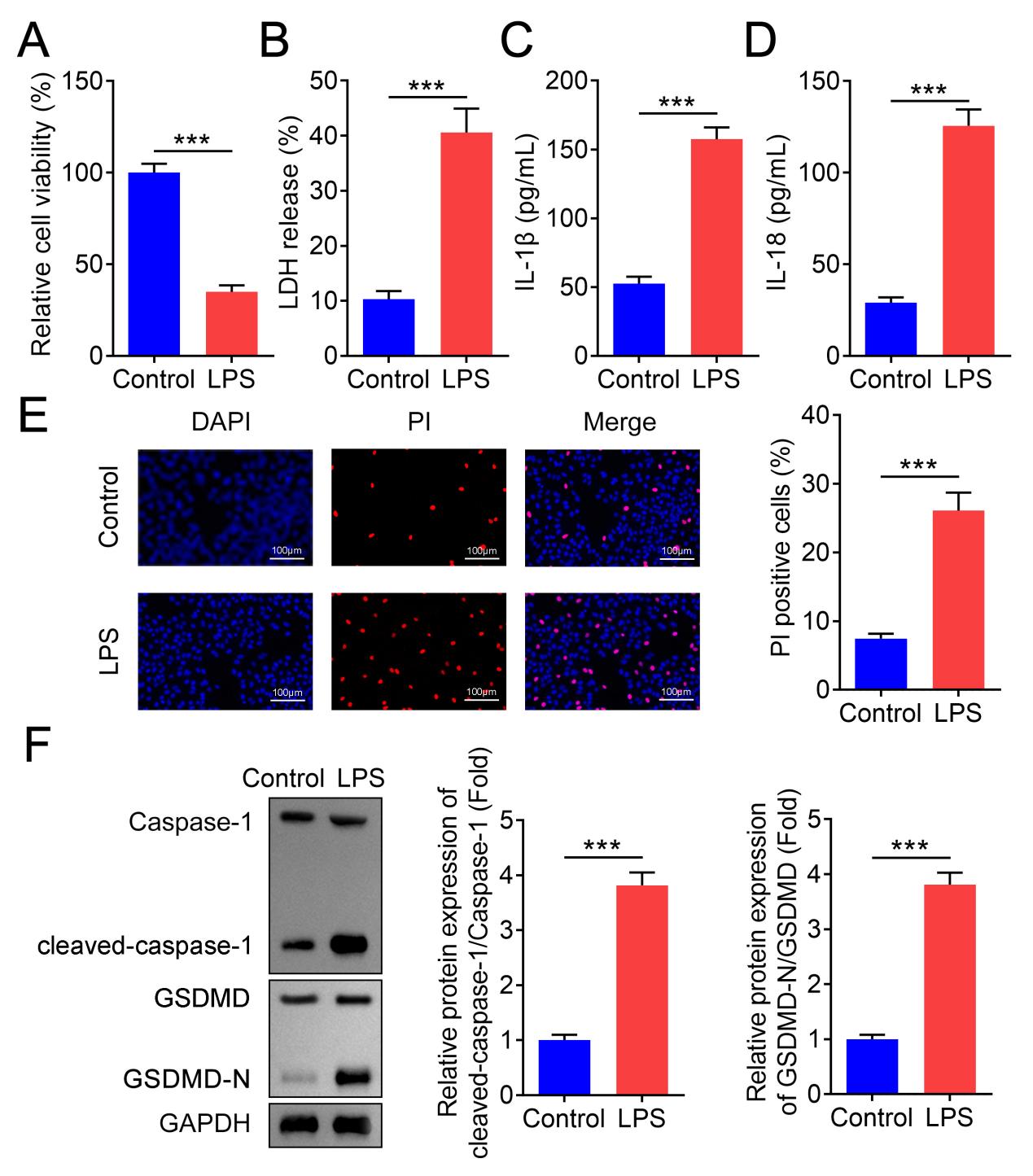

Supplement: Supplementary file 1 — Figure S1: The levels of O‐GlcNAc in mouse brain tissues in both PBS and MPTP group evaluated by western blotting method. Figure S2: LPS treatment induces pyroptosis of BV2 cells. [file JCMM-29-e70874-s001.docx]
